# Recent Advances in Repurposing Disulfiram and Disulfiram Derivatives as Copper-Dependent Anticancer Agents

**DOI:** 10.3389/fmolb.2021.741316

**Published:** 2021-09-17

**Authors:** Vinodh Kannappan, Misha Ali, Benjamin Small, Gowtham Rajendran, Salena Elzhenni, Hamza Taj, Weiguang Wang, Q. Ping Dou

**Affiliations:** ^1^ Research Institute in Healthcare Science, Faculty of Science and Engineering, University of Wolverhampton, Wolverhampton, United Kingdom; ^2^ Disulfican Ltd, University of Wolverhampton Science Park, Wolverhampton, United Kingdom; ^3^ Departments of Oncology, Pharmacology and Pathology, School of Medicine, Barbara Ann Karmanos Cancer Institute, Wayne State University, Detroit, MI, United States; ^4^ Centre for Interdisciplinary Research in Basic Sciences, Jamia Millia Islamia, New Delhi, India

**Keywords:** disulfiram, diethyldithiocarbamate, copper, disulfiram copper complex, drug repurposing, drug-delivery-system, cancer, cancer stem cells

## Abstract

Copper (Cu) plays a pivotal role in cancer progression by acting as a co-factor that regulates the activity of many enzymes and structural proteins in cancer cells. Therefore, Cu-based complexes have been investigated as novel anticancer metallodrugs and are considered as a complementary strategy for currently used platinum agents with undesirable general toxicity. Due to the high failure rate and increased cost of new drugs, there is a global drive towards the repositioning of known drugs for cancer treatment in recent years. Disulfiram (DSF) is a first-line antialcoholism drug used in clinics for more than 65 yr. In combination with Cu, it has shown great potential as an anticancer drug by targeting a wide range of cancers. The reaction between DSF and Cu ions forms a copper diethyldithiocarbamate complex (Cu(DDC)_2_ also known as CuET) which is the active, potent anticancer ingredient through inhibition of NF-κB and ubiquitin-proteasome system as well as alteration of the intracellular reactive oxygen species (ROS). Importantly, DSF/Cu inhibits several molecular targets related to drug resistance, stemness, angiogenesis and metastasis and is thus considered as a novel strategy for overcoming tumour recurrence and relapse in patients. Despite its excellent anticancer efficacy, DSF has proven unsuccessful in several cancer clinical trials. This is likely due to the poor stability, rapid metabolism and/or short plasma half-life of the currently used oral version of DSF and the inability to form Cu(DDC)_2_ at relevant concentrations in tumour tissues. Here, we summarize the scientific rationale, molecular targets, and mechanisms of action of DSF/Cu in cancer cells and the outcomes of oral DSF ± Cu in cancer clinical trials. We will focus on the novel insights on harnessing the immune system and hypoxic microenvironment using DSF/Cu complex and discuss the emerging delivery strategies that can overcome the shortcomings of DSF-based anticancer therapies and provide opportunities for translation of DSF/Cu or its Cu(DDC)_2_ complex into cancer therapeutics.

## Introduction

Cancer is a prominent cause of death worldwide which places an increasing burden on health and socioeconomic systems. According to the estimates provided by World Health Organization (WHO) in 2019, cancer is already the first or second cause of death before the age of 70 in 112 of 183 countries and its prevalence is expected to increase (Sung et al., 2021). It is predicted that ageing combined with population growth will result in an increase in the annual number of new cancer cases from 18.1 million in 2018 to 28.4 million in 2040. This increase will be severe in low- and middle-income countries, which are least prepared to the challenge (Wild, 2019; Sung et al., 2021).

In addition to surgery and radiation, chemotherapy which uses broad-spectrum cytotoxic drugs that do not differentiate cancer cells from normal cells, remains as the main arm of cancer treatment, despite its toxicity and significant side effects (Bedard et al., 2020). The discovery of potent metal-based chemotherapeutic drugs such as cisplatin, oxaliplatin and carboplatin paved the way for the development of a new class of inorganic metal-based chemotherapeutic agents (Rottenberg et al., 2021). These metal drugs bind to DNA and block the transcription and replication that initiate the process of apoptosis in cancer cells. Platinum drugs are commonly used alone or in combination with other chemotherapeutic agents to treat various malignant diseases such as testicular, lung, breast, ovarian, colon, head and neck cancer (Ndagi et al., 2017; Rottenberg et al., 2021). However, their use is still limited due to innate or acquired resistance in cancer cells and the undesirable side effects associated with their toxicity (Marine et al., 2020).

By the turn of the millennium, insights into the genomic landscapes of cancers led to the development of drugs that selectively target cancer cells while sparing normal cells, hence having high potency and fewer adverse effects (Druker et al., 2001; Cohen et al., 2021). Over the past two decades, over 40 receptor tyrosine kinase inhibitors (TKIs) have been approved by the US FDA as targeted drugs for cancer treatment (Cohen et al., 2021). Although TKIs such as imatinib have shown promising outcomes in some cancers like chronic myeloid leukaemia, the problems associated with emergence of resistance to treatment, tumour relapse and undesirable side effects and toxicity, remain as the major challenges for their use in cancer patients (Kobayashi et al., 2005; Marine et al., 2020). More recently, it has been identified that even the state-of-the-art immunotherapies, such as immune check-point inhibitors and adoptive T cell therapies which revolutionised the treatment of advanced cancers, are no exception to innate and acquired resistance mechanisms in cancers (Restifo et al., 2016; Hiam-Galvez et al., 2021). Moreover, the current growth in the cost of many targeted therapies for cancer discourages their use in clinical setting in terms of cost benefit ratio evaluation by healthcare agencies (Saluja et al., 2018; Leighl et al., 2021).

Owing to the clinical success of platinum drugs in a wide range of cancers and the requirement for reduction of treatment costs, there has been renewed interest in the development of novel metallodrugs in the field of inorganic drug development (Ndagi et al., 2017; Rottenberg et al., 2021). While the question of how cancer cells develop resistance to various targeted therapies still remains, there has been better understanding of the fundamental roles that several metal ions play in biological processes and their interaction with biomolecules. Recently, several non-platinum metallodrugs such as copper(II) (Cu), gold(I)-, ruthenium(III), gallium(III), cobalt(II)- and nickel(II)-based compounds have been investigated for their anticancer potential (Frezza et al., 2010; Chitambar, 2012; Lee et al., 2020; Yusoh et al., 2020). Each transition metal has unique ligand exchange kinetics and redox potentials and hence the choice of the metal centre and ligand design play a crucial role in the therapeutic effects of the metallo-complex compounds. Among transition metals, Cu has been widely proven to be used in development of therapeutic compounds due to the interesting anticancer properties and ease of handling to reduce side effects (Shanbhag et al., 2021). The nature of Cu ligands and the planar square geometry exhibited by Cu complexes is ideal for causing DNA specific damage very similar to that of platinum compounds (Lelievre et al., 2020). Cu plays crucial roles in many biological processes such as free radical detoxification, mitogenic signalling, immunomodulation, peptide processing, cellular respiration, and proliferation, and hence it is an indispensable micronutrient for all living organisms (Michniewicz et al., 2021; Shanbhag et al., 2021). However, in pathological conditions like cancer, excessive levels of Cu have been associated with enhanced proliferation, angiogenesis, and metastasis of cancer cells leading to cancer progression. Given the important role of Cu in the onset and progression of cancer, targeting Cu homeostasis has emerged as a novel strategy in the treatment of cancer (Li, 2020; Michniewicz et al., 2021).

Considering the reduction of development costs and affordability as a priority, repurposing old metal binding drugs to form complexes with Cu to selectively target cancer cells has been rigorously investigated in the past years (Bertolini et al., 2015; Saluja et al., 2018). One such candidate is Disulfiram (DSF), an old FDA approved anti-alcoholism drug with excellent metal chelation ability and demonstrated anticancer potential in a wide range of cancers in preclinical settings (Lu et al., 2021).

Here, we review the roles for Cu in cancer progression and the potential of harnessing copper homeostasis pathways in cancer cells with the Cu-binding activity of DSF and its derivatives, with a focus on recent developments in the translation of DSF for cancer therapy.

## Role of Copper in Cancer Progression

Dietary supplementation and absorption in the stomach, duodenum and upper small intestine is the main source of Cu in humans. Most of the absorbed Cu is stored in the liver and the Cu concentration in serum is approximately 12.0–25.0 μmol/L and varies in tissues between 15.0 and 180.0 μmol/g (Table 1). In physiological conditions the transport of Cu is strictly controlled. Cu is always bound to proteins to prevent excess redox activity and its distribution is precisely regulated by several cellular mechanisms. In mammalian cells, intracellular Cu intake is regulated by copper transport protein 1 (Ctr1) and Cu enters the cells in reduced form [Cu(I)]. Once inside the cell, Cu is bound to Cu chaperons or glutathione (GSH) and trafficked to metallothionein or distributed to specific compartments of the cells. Despite being an essential micronutrient, free Cu can induce toxicity to cells (Wee et al., 2013; Michniewicz et al., 2021).

**TABLE 1:** Tissue and serum levels of copper in normal and cancer patients (***p ≤ 0.001, **p ≤ 0.01, *p ≤ 0.05).

Cancer type	Number of Samples		Tissue Copper levels (µg/g) (mean ± SD		Reference
	Normal tissue	Tumor tissue	Normal tissue	Tumor tissue	
Breast	25 (matched pair)	25 (matched pair)	9.3 ± 2.3	21.0 ± 10.7^***^	Rizk and Sky-Peck (1984)
	68 (matched pair)	68 (matched pair)	6.13 ± 4.32	11.31 ± 7.83^**^	Kuo et al. (2002)
Gynaecologic organs	47	53	1.26 ± 0.45	2.16 ± 0.6^**^	Margalioth et al. (1983)
Ovarian			0.3 ± 0.1	0.7 ± 0.3^**^	Yaman et al. (2007)
Lung	100	64	5.08 ± 1.09	8.23 ± 4.88^**^	Diez et al. (1989a)
Stomach	47	53	1.44 ± 0.38	2.09 ± 0.52^*^	Margalioth et al. (1983)
	4, 14 (matched pair)	14 (matched pair)	1.1 ± 0.4	1.7 ± 0.1^**^	Yaman et al. (2007)
Gallbladder	30 (sex matched)	30	16.30 ± 7.28	21.01 ± 6.67^**^	Basu et al. (2013)
Colorectal cancer	50	50	0.12 ± 0.05	0.17 ± 0.08	Sohrabi et al. (2018)
	**Number of Samples**		**Serum levels of copper (µg/dl) (mean ± SD**		
	**Normal level**	**Cancer patients**	**Normal level**	**Cancer patients**	
Breast	21	8	96 ± 11.52	152.96 ± 21.76^*^	Zowczak et al. (2001)
	20 (normal)	56 (carcinoma) 32 (benign)	101.62 ± 10	115.9 ± 14.4^**^	Feng et al. (2012)
	54	54	109.56 ± 30.71	202.21 ± 89.18^***^	Pavithra et al. (2015)
	25	68	96.5 ± 7.3	125.2 ± 15.0^**^	Kuo et al. (2002)
	30	30	65.2 ± 15.0	95.3 ± 4.9^**^	Adeoti et al. (2015)
	84	88	113.2 ± 13.6	137.2 ± 36.2^***^	Ding et al. (2015)
Leukaemia	20	49	86.7 ± 25.3	139.9 ± 51.2^***^	Zuo et al. (2006)
	40	42	104.7 ± 0.16	122.6 ± 0.18^***^	Demir et al. (2011)
	40	50	124.22 ± 10.36	146.25 ± 10.47^**^	Mehdi et al. (2015)
	21	12	114 ± 29	328 ± 74^**^	Carpentieri et al. (1986)
	13	14	126.8 ± 21.2	256.8 ± 73.8^***^	Delves et al. (1973)
Lung	53	109	101.1 ± 7.96	128.6 ± 6.85^*^	Oyama et al. (1994)
	154	154	128.5 ± 5.23	162.4 ± 8.18^**^	Jin et al. (2011)
	100	20 (benign) 64(malignant)	112.5 ± 17	150.3 ± 33.3^***^	(Diez et al. 1989a; Diez et al. 1989b)
Oral	66	60	114.20 ± 38.69	209.85 ± 160.28^***^	Baharvand et al. (2014)
	30	40	105.5 ± 18.81	141.99 ± 21.44^***^	Tiwari et al. (2016)
Gastrointestinal	21	26	96 ± 11.52	128.64 ± 20.48^*^	Zowczak et al. (2001)
Thyroid	20	30	75.5 ± 8.1	85.4 ± 8.7^*^	Baltaci et al. (2017)
Gall bladder	30	30	125.97 ± 38.93	159.19 ± 24.04^***^	Basu et al. (2013)
Prostrate	30	22	97 ± 22	169 ± 31^***^	Saleh et al. (2020)
Colorectal	966 (matched pair)	966 (matched pair)	135.8 ± 30.5	138.6 ± 30.8^**^	Stepien et al. (2017)
Gynaecological	20	100	44 ± 0.2	179 ± 8.3^**^	Cetinkaya et al. (1988)

Many studies have indicated that the levels of Cu in both serum and tumour tissues are remarkably elevated in cancer patients in comparison to healthy individuals as summarised in Table 1. Significantly higher Cu levels in serum have been associated with multiple cancers such as breast, cervical, ovarian, lung, gastric, bladder, thyroid, oral, pancreatic, head, and neck cancer (Li, 2020; Shanbhag et al., 2021). In some malignancies like colorectal cancer and breast cancer, elevated Cu levels are strongly correlated with the stage and progression of the tumours (Gupta et al., 1993; Sharma et al., 1994). On the other hand, in haematological cancers, a reduction in Cu levels was observed during periods of remission in patients, indicating that the distribution of Cu is altered in cancer patients (Kaiafa et al., 2012).

Although the underlying mechanisms alter the Cu levels in the serum of cancer patients remain unclear, the role played by Cu in etiology and progression of tumours has been widely studied in the past two decades. Exposure to high levels of Cu has been linked with pancreatic neuroendocrine tumours and prostate cancer (Ishida et al., 2013; Vella et al., 2017). As a metal able to induce redox reactions, Cu is capable of generating reactive oxygen species (ROS) in the intracellular environment that leads to activation of various pro-oncogenic signalling mechanisms that enhances the proliferation of cancer cells (Prasad et al., 2017). It has been recently shown that Cu is involved in the regulation of MAP kinase pathway and oncogenic BRAF signalling that regulates cell proliferation, differentiation and motility, by interacting with MEK-1 protein, a key penultimate kinase in the MAPK pathway. MEK-1 contains a high affinity Cu binding site, which, upon binding of Cu will stimulates the MEK-1 dependent phosphorylation of ERK1/2, ultimately promoting tumour proliferation (Brady et al., 2014).

Recently it has been shown that enhanced levels of Cu upregulate the expression of programmed death ligand 1 (PD-L1) in tumour cells and modulate the signalling pathways that mediates the PDL-driven immune escape by cancer cells (Voli et al., 2020). It has also been shown in the same study that chelators of Cu enhanced the ubiquitin-mediated degradation of PD-L1 and resulted in the infiltration T lymphocytes and natural killer cells in the tumour site, thereby suppressing tumour growth and improving survival in mouse models (Voli et al., 2020).

Cu has the ability to switch on many pro-angiogenic responses and aid the proliferation and mobility of endothelial cells, which initiates angiogenesis that provides oxygen and essential nutrients to tumours (Finney et al., 2009; Xie and Kang, 2009). Cu is considered as a key angiogenic messenger, because it can stabilise nuclear hypoxia inducible factor-1 (HIF-1), leading to expression and activation of several angiogenic factors including fibroblast growth factor (FGF), vascular endothelial growth factor (VEGF), tumour necrosis factor alpha (TNF-α), interleukins 6 and 8 (IL-6, IL-8), and fibronectin (Martin et al., 2005; Feng et al., 2009; Rigiracciolo et al., 2015). Cu also promotes the remodelling of extracellular matrix through activation of the Cu-dependent enzyme lysyl oxidase (LOX). Similarly, another Cu-dependent protein known as superoxide dismutase-1 (SOD1) which is a regulator of vasoconstriction and endothelial function, is overexpressed associated with elevated Cu levels in cancers, which eventually stimulates VEGF production and enhances FGF-induced angiogenesis and tumour development, while a decrease in SOD1 activity has been shown to impair angiogenesis (Xiao and Ge, 2012; Shanbhag et al., 2019). In addition to the above, Cu is also shown to enhance the metastatic potential of cancers through activation of metabolic proteins such as (LOX) and lysyl oxidase-like (LOXL) which are involved in remodelling extracellular matrix and creation of pre-metastatic niches that harbours metastatic cancer cells (Salvador et al., 2017). Many recent studies have shown that there are other Cu-dependent or Cu-binding proteins such as mediator Of Cell Motility 1 (MEMO1), copper metabolism MURR1 domain-containing protein 1 (COMMD1), Antioxidant Protein 1 (ATOX1) and secreted protein acidic and rich in cysteine (SPARC) which are involved in the process of cell migration and invasion by modulation of cytoskeleton, extracellular remodelling or by formation of adhesion sites (MacDonald et al., 2014; Nagaraju et al., 2014; Blockhuys et al., 2020).

## Copper Chelators and Ionophores in Cancer

Cu homeostasis in cancers is emerging as an attractive target for anti-cancer drug development. There are two main approaches that have been tested in both preclinical and clinical conditions. The first approach involves using Cu chelators which reduce the bioavailability of Cu by directly binding to Cu; the second strategy is to use Cu ionophores that can increase the intracellular levels of Cu and therefore exert antitumor effects through ROS production, proteasome inhibition, and apoptosis induction (Li, 2020). Cu chelators such as D-penicillamine (D-pen), tetrathiomolybdate (TM), and trientine, are being widely investigated for their anticancer activity both *in vitro* and *in vivo* as well as in clinical trials (Wadhwa and Mumper, 2013). D-pen is a very strong metal chelator identified in early 1950s. D-Pen has the ability to remove Cu *in vivo* and the combination of D-pen and Cu could lead to cell death in endothelial cells and lymphocytes possibly as a result of ROS production and LOX and ICAM inhibition, eventually suppressing tumour growth and vascularization (Lipsky and Ziff, 1978; Starkebaum and Root, 1985; Brem et al., 1990; Gupte and Mumper, 2007). TM is another highly specific chelator of Cu and its anticancer activities has been studied well since the 1990s. TM has been demonstrated to inhibit angiogenesis in tumours by targeting multiple pathways including the suppression of NF-κB, HIF1α, LOX and SOD1 activities (Brewer, 2003). More recently, it has been shown that TM can inhibit MEK1/2 kinase activity by reducing the levels of intracellular Cu, and eventually suppressing BRAF-driven tumorigenesis in melanoma, thyroid and colon cancers (Kim et al., 2020; Xuefeng et al., 2020). TM can also enhance the cytotoxicity of BRAF-specific inhibitors like sorafenib. Trientine is another Cu chelator developed in the late 1960s, which has weaker activity than TM and D-Pen but is more tolerable than the other two in patients. The polyamine structure of trientine promotes Cu elimination *via* urinary excretion. It has been shown that trientine can inhibit endothelial cell proliferation by reducing the levels of IL-8 and CD31 expression (Moriguchi et al., 2002; Yoshiji et al., 2005).

While more research and encouraging clinical trials are ongoing for Cu chelators, Cu ionophores are also being widely studied for their anticancer activities. The mode of action of Cu ionophores is highly dependent on Cu as the compounds on their own have very little anticancer effect. Examples of Cu ionophores include Clioquionol, docosahexaenoic acid, thiosemicarbazone and DSF. Mechanistically these compounds exert their anticancer activities by elevation of extracellular and intracellular ROS, DNA damage and proteasome inhibition (Cater and Haupt, 2011; Yu et al., 2017; Sun et al., 2020). Clioquinol was shown to induce caspase-dependant apoptosis, but its clinical use has been discontinued due to its neurotoxicity (Cater and Haupt, 2011). Preclinical studies using analogues of Cloquionol are ongoing with different routes of administration for better anticancer activity and safety (Wehbe et al., 2018).

Among these compounds, DSF is the most extensively studied compound. DSF is also an attractive compound because of its established safety profile, easy availability, low cost, and less adverse effects than anticancer drugs. DSF can specifically carry Cu ions into tumour tissue, thereby preventing nonspecific interactions (Ekinci et al., 2019). Recently, some studies investigated the action of Cu complexes formed by some Cu-binding compounds such as DSF derivatives and its metabolites. Many clinical trials of DSF and Cu in combination with or without conventional anticancer drugs are under way which will be reviewed and summarised in the later part of this review.

## Drug Repurposing and Disulfiram

From boosting immune system to targeting key molecules and developing personalized therapy cancer drug development is at its exciting phase today. New drug development is an expensive process, costing over $1.5 billion and taking approximately 15 yr to complete the process, however, there is a very high failure rate of up to 95% (Bertolini et al., 2015). Costs of this magnitude arrest research and development, forcing pharmaceutical developers to pitch for more money or suspend development altogether. Consequently, some of these new therapies reach price tags of more than $100,000 per patient per year, but still has no “meaningful benefit” (Saluja et al., 2018; Leighl et al., 2021). The prices of cancer drugs have increased 10% every year between 1995 and 2015 due to the escalated expense of R&D in the pharmaceutical industries (Leighl et al., 2021). In recent years there has been an increasing appreciation of the potential of repurposing known drugs like DSF, as an anticancer treatment. The approach is pointed towards developing cheaper alternative over the expensive ineffective drugs and reduce the burden on healthcare systems, while providing effective treatment options for thousands of patients diagnosed every year (Lu et al., 2021).

### Disulfiram

DSF or Tetraethylthiuram disulfide was developed in the late 19th century for the industrial production of rubber. The intolerance to alcohol in factory workers exposed to DSF was first observed in the late 1930s and it was not until the late 1940s its potential as an anti-alcoholism medicine was recognised (Suh et al., 2006). Further its potential as a scabicide and vermicide medicine was recognised in the 1947, where DSF was utilised as an effective chelator of iron, copper, and zinc that caused toxicity in the parasites by damaging the copper-containing respiratory enzymes (Gordon and Seaton, 1942). Again, it was observed that upon alcohol intake when using this drug, it produced adverse side effects. In 1948, it was identified that the alcohol-aversive effects of DSF was due to the accumulation of acetaldehyde in patients (Hald and Jacobsen, 1948). Subsequently, DSF was established as a commercially available FDA anti-alcoholism drug in 1951. Overall, DSF has been in use in the clinic for over 70 yr in various fields such as parasitology, infectious diseases, virology and oncology (Suh et al., 2006).

### Chemistry and Pharmacokinetics of Disulfiram

Disulfiram is an off-white crystalline powder that is insoluble in water but has variable solubilities in organic solvents like ethanol and ether. DSF is a relatively small molecule with a molecular weight of 296.54, density of 1.30 and a melting point of 70°C−72°C. DSF has oxidative biotransformation properties yielding sulphate, methanethiol and formaldehyde metabolites (PubChem CID3117).

**FIGURE 1:**
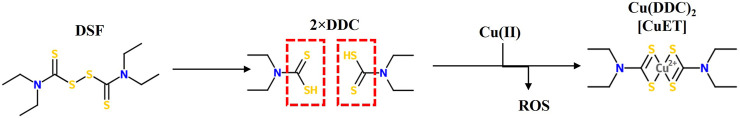
The structure of Disulfiram (DSF), its metabolite diethyldithiocarbamate (DDC) and its complex with copper Cu(DDC)_2_ or (CuET). Intact thiol groups in DS or DDC are indispensable for the chelation of DS or DDC with copper a reaction that results in the generation of reactive oxygen species (ROS).

Both DSF and its metabolite diethyldithiocarbamate (DDC) are stable in basic environments, but unstable in acidic conditions. Upon ingestion, more than 99% of DSF is quickly converted to its corresponding thiol metabolite DDC in the acidic environment of the stomach. Both forms are rapidly absorbed through the gastrointestinal mucosa into the portal circulation and enriched in liver where both DSF and DDC are rapidly metabolised and degraded. DDC, due to its hydrophilic polar nature, is easily reduced to diethylamine (DEA) and carbon disulphide (CS2). CS2 is reactive with endogenous nucleophilic groups including thiols, amino acids and proteins and can be oxidized to form carbonyl sulphide (COS). COS can be further oxidised to form carbon dioxide and sulphur radicals, resulting in a permanent inhibition of microsomal mono-oxygenases. Further, CS2 can be involved in re-synthesis of DDC by reacting with DEA *in vivo*, that might explain the potential intolerance to alcohol for a longer period of time (Eneanya et al., 1981; Johansson, 1992; Petersen, 1992). Both DSF and DDC are strong chelators of divalent transition metal ions and forms stable complexes with heavy metal ions (Figure 1). The metal complex, Cu (DDC)_2_, is a dark precipitate which is more stable in acidic environments and the neutral and hydrophobic nature of Cu(DDC)_2_ enables its systemic absorption to take place along the upper GI tract (Johansson, 1992). The intact thiol group in DSF and DDC is essential and indispensable for them to chelate divalent transition metal ions.

DSF, after being absorbed into circulation is immediately broken into DDC monomers by the glutathione reductase system in red blood cells. Free DDC quickly binds to thiols of proteins, specifically to albumins, to form disulfides. DDC is also a substrate for Phase II metabolism that is mediated by an S-methyl-transferase, resulting in the formation of S-methyl diethyldithiocarbamate (S-Me-DDC) and S-glucuronide of DDC. Oxidative biotransformation of S-Me-DDC forms S-diethylthiomethylcarbamate (S-Me-DTC), which could be further oxidized to S-Me-DTC-sulphoxide and S-Me-DTC-sulphone metabolites, respectively (Eneanya et al., 1981). All these methylated metabolites are also involved with the inhibition of isozymes of aldehyde dehydrogenases (ALDH) resulting in the disulfiram-ethanol reaction (DER) (Yourick and Faiman, 1989; Hart and Faiman, 1992; Mays et al., 1996; Lipsky et al., 2001). The methylation and glucuronidation reactions of DSF and DDC will block the key reactive thiol group and abolish their metal chelating activity.

### Pharmacodynamics of Disulfiram

Ethanol metabolism in the liver predominantly relies on the action of ALDH isozymes and a minor amount of ethanol is metabolised through P450 mono-oxygenases and catalases. Upon intake of alcohol, ethanol oxidation produces acetaldehyde which is further oxidised by ALDH to form acetate. Two important ALDH isoenzymes involved in this process are ALDH1 and ALDH2 (Johansson, 1992; Veverka et al., 1997). Sulphoxide, sulphone and the Me-DTC metabolites of DSF inhibit ALDH1 and ALDH2 through the formation of a covalent adduct, likely with the cysteine residue present at the active site of these enzymes, causing a permanent non-reducible, thiol-resistant reaction, so that this inhibitor is covalently bound into the active site of the enzyme (Hellstrom et al., 1983). This causes a rise in blood acetaldehyde concentrations and results in severe unpleasant side-effects such as hyperventilation, dyspnoea, hypotension, tachycardia, chest pain causing ischemia, vertigo, nausea, vomiting and headaches within 15 min on ingestion known as DER (Hellstrom et al., 1983; Petersen, 1992).

In addition to ALDH, DSF is also known to inhibit other enzymes involved in vital metabolic pathways such as glycolysis, tricarboxylic acid cycle, pentose phosphate shunt, glutathione system, and catecholamine synthesis through S-S linkage or by chelation of copper or zinc from the active sites of the enzymes (Eneanya et al., 1981). DSF can inhibit glyceraldehyde-3-phosphate dehydrogenase, Fructose-l,6-diphosphate dehydrogenase and succinic dehydrogenase which are key enzymes involved in the mitochondrial respiratory metabolism and can also interfere with the normal oxidative phosphorylation by blocking NAD+-dependent mitochondrial oxygen consumption. Both DSF and DDC can inhibit dopamine-β-hydroxylase, an enzyme that converts dopamine to norepinephrine catecholamine synthesis (Marselos et al., 1976; Eneanya et al., 1981).

### Anticancer Mechanisms of Disulfiram

**FIGURE 2:**
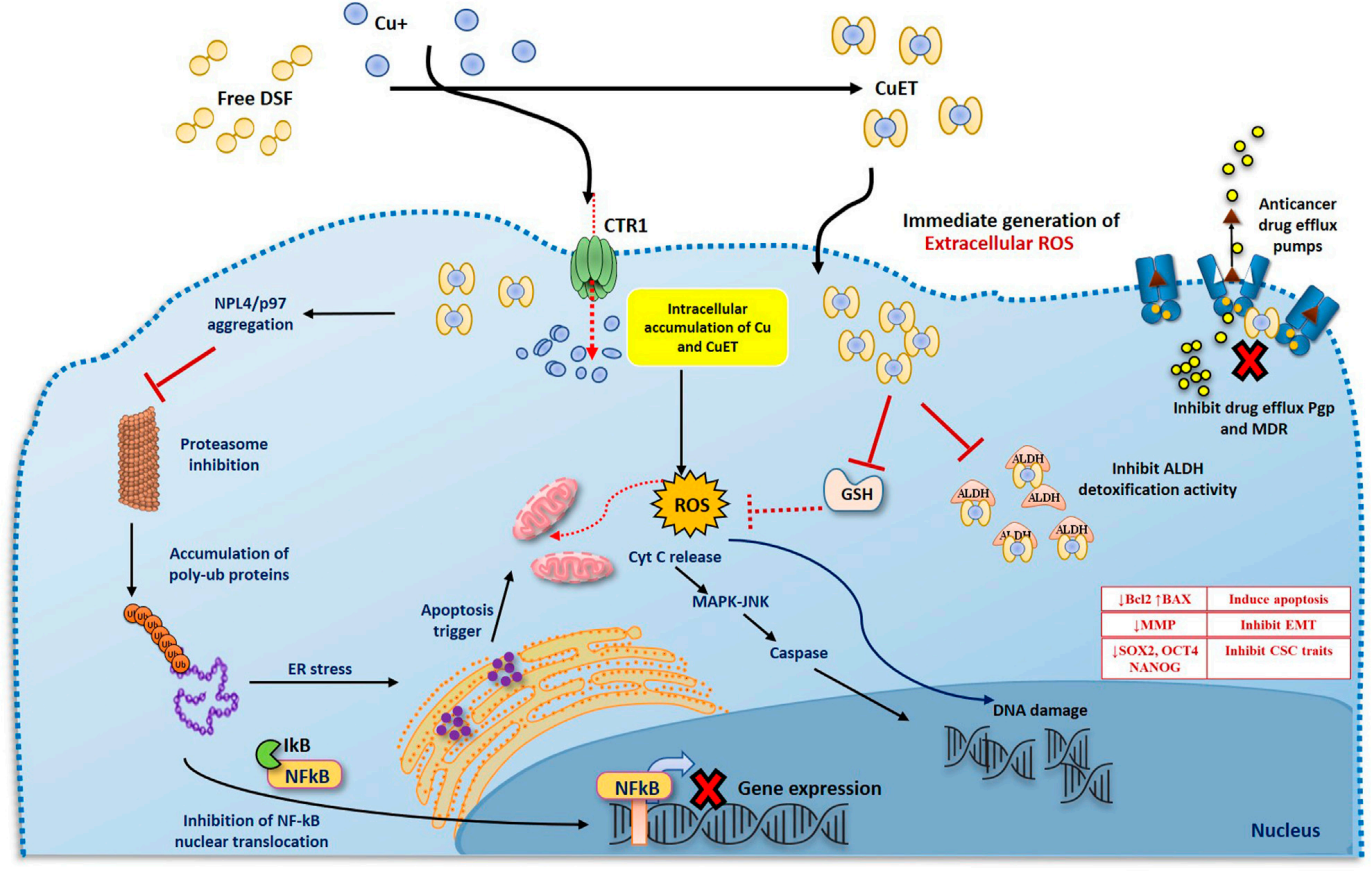
Two phases anticancer model of DS and DDC plus copper induced cancer cell death. 1. DS or DDC and copper contact and react extracellularly. The reaction will generate ROS extracellularly which induce cancer cell apoptosis. Due to the extremely short lifespan of ROS in tissue, the reaction must be taken place adjacent to cancer cells, in order to effectively target cancer cells. 2. The reaction-generated compound, CuDDC_2_ (CuET), can easily penetrate cancer cells and trigger intracellular ROS generation and induce cancer cell apoptosis.

The anticancer activity of DSF has been demonstrated in various cancer cells models and is shown to be strongly dependent on formation of Cu(DDC)2 complex with divalent metal ions such as Cu. In *in vitro* conditions, a mixture of DSF and Cu immediately results in a highly oxidized intermediate form of DDC termed as bis(dialkyliminium)-tetrathiolane dication (Bitt-42+), followed by subsequent spontaneous decomposition of small amount of DSF to its anionic chelate form DDC, which on further redox reaction with Cu2+ forms a stable complex Cu(DDC)2. This redox reaction and the Fenton chemistry involved in the formation of Cu(DDC)2 complex results in the generation of ROS, which induces apoptosis in cancer cells. DSF/Cu, has been shown to be cytotoxic to cancer cells and has the ability to eradicate cancer stem cell (CSC) populations in various cancers with little or no toxicity to normal cells (Liu et al., 2012; Liu et al., 2014; Xu et al., 2017; Yip et al., 2011). DSF/Cu also reverses acquired and hypoxia induced anticancer drug resistance and has been shown to potentiate anticancer drug-induced apoptosis in colon, breast, lung, liver and brain cancers (Ekinci et al., 2019). There are number of underlying mechanisms by which DSF act as an anticancer drug (Figure 2). It can either show cytotoxic effects itself or act as an adjuvant by sensitizing the cancer cells to many first line chemotherapeutic drugs (Wang et al., 2003; Triscott et al., 2012; Liu et al., 2013; Schmidtova et al., 2019; Lu et al., 2021).

### Disulfiram-induced Cell Death

**FIGURE 3:**
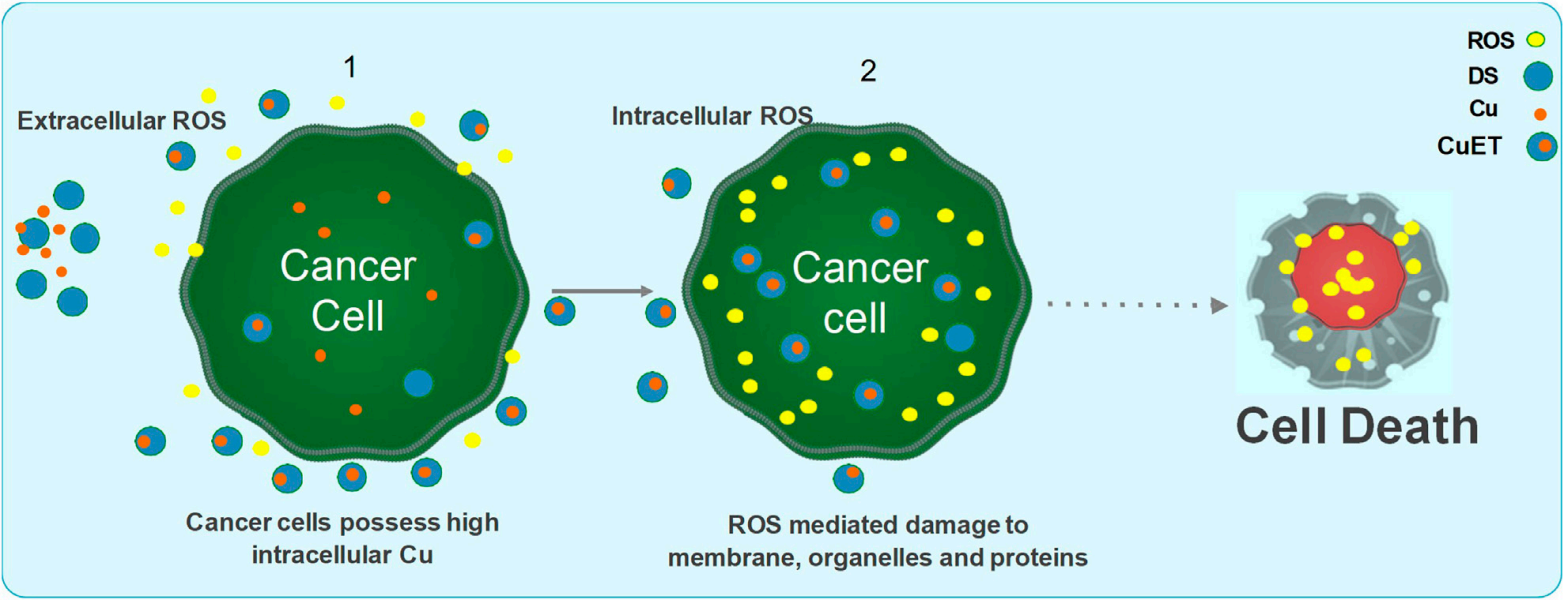
Mechanism of action of DSF/Cu induced anticancer activity. DSF and Cu reaction generates extracellular ROS and damage the membrane proteins. The formation of CuET complex outside the cell and transportation of CuET through lipid bilayer, in addition to increased influx of Cu *via* the CTR1 transporter, further triggers the intracellular ROS mediated mitochondrial damage and DNA damage leading to apoptosis *via* the MAPK pathway and JNK activation. CuET also inhibits the proteasome activity *via* the NPL4/p97 segregase pathway leading to inhibition of NFkB pathway. DSF and CuET further inhibits ALDH mediated ROS detoxification and Pgp mediated drug efflux mechanisms. Collectively, all the above negatively effects the cancer cell survival and maintenance of stemness and resistance thereby sensitizing the cells to ROS and anticancer drug mediated damage, eventually leading to apoptosis.

The anti-cancer mechanism of DSF appears to be Cu-dependent, as this plays a role in redox reactions. As cancer cells contain high amounts of Cu through the trans-membrane Cu transporter Ctr1 transportation, DSF can form a complex with Cu, enabling it to penetrate into cancer cells. This enables DSF to specifically target these cells and not normal healthy cells that express low levels of Cu (Liu et al., 2012). Reaction between DSF, DDC and Cu results in the generation of extracellular ROS, which in turn induce apoptosis in cancer cells (Lewis et al., 2014; Tawari et al., 2015). It is also demonstrated that the metabolite of DSF, DDC and its copper complex Cu(DDC)_2_ get accumulated in the cancer cells and induce ROS generation leading to apoptosis in cancer cells (Guo et al., 2010; Yip et al., 2011; Tawari et al., 2015; Lu et al., 2020; Majera et al., 2020; Yoshino et al., 2020). Generation of both extracellular and intracellular ROS is completely relied on the intact thiol group which chelates copper (Figure 3).

DSF has been shown to inhibit superoxide dismutase and competes with glutathione reductase causing an imbalance is the ROS scavenging mechanisms of cancer cells. Further, DSF, being a specific inhibitor of ALDH, blocks the ROS scavenging and detoxification mediated by ALDH isozymes. Increased ROS levels and inhibition of ROS scavenging mechanisms render the cells vulnerable to subsequent oxidative stress and further DNA damage which triggers the DNA-damage response mechanisms. DSF associated with Cu is shown to down-regulate the expression of several genes involved in DNA repair pathways. Thus, the ROS generated by DSF/Cu or Cu(DDC)_2_ is sustained in the cells leading to high levels of DNA damage that induce mitochondrial pore opening and apoptosis *via* the MAPK trigger (Cen et al., 2004; Morrison et al., 2010; Tawari et al., 2015). Apoptosis induced by ROS is highly dependent on persistent activation of pro-apoptotic MAPK pathway which activates the mitochondrial pro-apoptotic proteins by phosphorylation (Junttila et al., 2008; Xu et al., 2020b). It was found that in breast cancer cells treated by DSF/Cu complex, there was persistent activation of MAPK pathway which were then directed to ROS-induced apoptosis. Also, the cytotoxic effect of DSF/Cu complex was reduced by MAPK pathway inhibitors, indicating the role of MAPK pathway in ROS-induced apoptosis (Yip et al., 2011). Chiba et al. (2014) demonstrated that tumor-initiating hepatocellular carcinoma cells were significantly reduced by DSF-induced apoptosis *via* activation of MAPK pathway (Chiba et al., 2014). Nasopharyngeal cancer cells were also eradicated by DSF/Cu complex through increasing the ROS levels and activating the apoptosis-related MAPK pathway (Li et al., 2020). Zhang et al. (2019) demonstrated that DSF/Cu complex induced the autophagy-dependent apoptosis in human pancreatic and breast cancer cells through the activation of ER-stress (Zhang et al., 2019). DSF/Cu complex can also induce apoptosis *via* activation of chloride channel-3 (ClC-3). These ClC-3 channels are highly functional and over-expressed in cancer cells as compared to normal cells, and DSF/Cu complex is only able to trigger activation of over-expressed ClC-3 channels. Therefore, DSF/Cu complex specifically induce apoptosis in the cancer cells, through targeting over-expressed ClC-3 channels, without affecting the normal cells (Xu et al., 2019). In summary, the increased ROS production and subsequent cell death induced by DSF was found to be largely dependent on the presence of Cu at a required level.

### Disulfiram and Proteasome Inhibition

A functional proteasome system maintains the synchronized expression of cell cycle-regulatory and apoptosis-control proteins. Hence the proteasome-mediated protein degradation is an important cellular homeostatic system, and in general cancer cells are more sensitive to alterations in this proteasome inhibition than normal cells. Bortezomib was the first clinically approved 20S proteasome inhibitor drug used for its antitumor activity against several types of cancers (Adams, 2004; Papandreou and Logothetis, 2004; Kane et al., 2006). Proteasome inhibition activity of DSF was first reported by Lovborg et al. (2006) by showing its specific inhibition for 26S proteasome (Lovborg et al., 2006). Combination of DSF or metabolite DDC with Cu has been shown to be a potent inhibitor of the functional proteasomes in many cancers (Kona et al., 2011; Triscott et al., 2012; Brüning and Kast, 2014; Yoshino et al., 2020). Notably, DSF/Cu was shown to inhibit the proteasome activity in breast cancer models but not in normal breast cells. The inhibition of proteasome activity by DSF/Cu complex leads to the accumulation of poly-ubiquitinated proteins and cytotoxic protein aggregates of important proteins such as IκB, p27, Kip1 and c-Myc, which results in the inhibition of cell-cycle progression and subsequent apoptosis (Cvek and Dvorak, 2008). Studies from our group (Chen et al., 2006) showed that low concentrations of DSF can partially bind to the 20S proteasome molecule and inhibiting the chymotrypsin-like activity of the proteasome. It is also speculated that DSF inhibits POH1, a protein which plays an important role in deubiquitinating function of the proteasome lid rather than the actual core proteasome (Chen et al., 2006; Cvek and Dvorak, 2007; Gallery et al., 2007; Yu et al., 2007; Cvek et al., 2008; Yoshino et al., 2020).

The protein complex, nuclear factor-kappa B (NF-κB), has a close relationship to cancer cells by acting as a key regulator of multiple pathways necessary for survival, stemness, resistance, angiogenesis and metastasis (Cvek and Dvorak, 2007; Cvek and Dvorak, 2008). NF-κB is a transcriptional regulator of various genes and often expressed in high levels in cancer cells in comparison to normal cells. The activity of proteasomes is very important for activation of NF-κB pathway, where proteasomes are involved in degradation of the inhibitor-KB molecule (IkB), which eventually releases NF-κB p50/p65 heterodimer from the inhibitory complex to translocate into the nucleus and exert its function as transcriptional regulator (Verzella et al., 2020). There is a close relationship between chemoresistance and activation of NF-κB in cancer cells especially in response to DNA damage induced by anticancer drugs (Wang et al., 1999; Wang and Cassidy, 2003). If DSF/Cu blocks the proteasome system, IκB continuously inhibits NF-κB, preventing its nuclear translocation which eventually and favours apoptosis or sensitise cancer cells to anti-cancer drugs. In this regard, DSF played an important role demonstrated by Wang et al. (2003) that DSF when administered along with 5-fluorouracil, it significantly inhibited the activity of NF-κB and enhanced the apoptotic effect of 5-fluorouracil on colorectal cell lines, DLD-1 and RKO (Wang et al., 2003). Reversal of chemo-resistance by DSF through the inhibition of NF-κB was also observed in breast cancer cells and human glioblastoma cell lines (Yip et al., 2011; Liu et al., 2012; Liu et al., 2014). Similarly, DSF-copper complex has also been found quite effective in inhibiting the activity of NF-κB and reversing the chemo-resistance of colon and breast cancer cell lines against the anti-cancer drug gemcitabine (Guo et al., 2010). It has also been found to have cytotoxic effect against the leukaemia stem cells by simultaneously inducing the apoptosis through ROS-JNK pathway and inhibiting the pro-survival NF-κB pathway (Xu et al., 2017). NF-κB induces epithelial–mesenchymal transition EMT by the up-regulation of master-switch transcription factors for EMT (Chua et al., 2007; Julien et al., 2007; Huang et al., 2013) and stabilization of Snail which suppress the expression of adherent junction proteins (Wu et al., 2009; Yang et al., 2013). Therefore, DSF-copper complex can also suppress the EMT event in cancer by inhibiting the activity of NF-κB, clearly demonstrated by Li et al. (2018) in hepatocellular carcinoma (Li et al., 2018).

However, a recent study argued that the DSF/Cu complex (Cu(DDC)2 or CuET) is not a direct inhibitor of proteasomes, instead CuET targeted the p97-NPL4-UFDI pathway upstream to proteasome. It was shown that CuET binds to the NPL4, resulting in aggregation of NPL4 and deactivation of P97 segregase which leads to accumulation of misfolded proteins in the cells and ultimately cell death (Skrott et al., 2017). It was also described those cells treated with the CuET complex shows similar phenotypic characteristics as that of proteasome inhibitors, such as accumulation of poly-ubiquitinated proteins in the cytoplasm (Skrott et al., 2017).

The main target of CuET in p97 segregase pathway is NPL4, a key component of p97 segregase. The interaction of NPL4 and CuET results in the formation of protein aggregates and immobilization of otherwise very mobile NPL4-p97 complex which triggers the cellular heat shock response and endoplasmic reticulum (ER) stress/unfolded protein response, eventually leading to cell death (Ding and Zhu, 2018; Skrott et al., 2019). CuET can also induce replication stress *via* NPL4 targeting. Skrott et al. (2017) reported that in addition to NPL4/p97, the CuET-induced aggregates consist of several proteotoxic stress-related proteins, including HSP70, SUMO2/3, polyubiquitin chains, and TDP-43. In their further studies they found that ATR kinase, a key factor required for proper cellular response to replication stress, is also trapped and sequestered in the NPL4 aggregates (Skrott et al., 2017). It is known that ATR kinase dysfunction triggers the replication stress, therefore, one could say that ATR aggregation is the primary and/or major cause of the CuET-induced replication stress (Majera et al., 2020). CuET has high affinity for binding to NPL4 because NPL4 contains two zinc-finger domains: C-terminal NZF (NPL4-zinc-finger) and putative zf-NPL4, respectively, which bind bi-valent metals and metal complexes. It was observed that it is putative zf-NPL4 which binds with CuET as it might chemically resembles the metal complexes, which binds to zf-NPL4 (Skrott et al., 2017). Recently, a more detailed description about the interaction of DSF/Cu complex with NPL4 has come into light. It has been shown that NPL4 goes through three conformational states while in complex with p97. These conformational changes seem like a seesaw motion. This motion of NPL4 takes place due to its zinc finger motifs interaction with N domain of p97 and it is essential for the unfolding activity of p97 (Pan et al., 2021). It has been observed that DSF/Cu complex bypasses the copper transporter system of the cell membrane and release the cupric ions under oxidative conditions. These cupric ions interact with the zinc fingers of NPL4 and lock the conformational switch of NPL4/p97 complex essential for the unfolding activity of the p97, hence, inhibits the function of p97 (Pan et al., 2021).

The same group also discovered that cells lacking BRCA1 and BRCA2 were particularly sensitive to DSF/Cu or CuET treatment. CuET induced replication stress-associated DNA damage in several cancer cell lines and increased γH2AX, as an indicator for DNA double-strand breaks in these cells (DSB). Homologous recombination (HR), a DSB repair mechanism, requires BRCA1 and BRCA2 for its activity. Interestingly, a co-localization of ATR with immobilized NLP4 aggregates was found after CuET treatment. Moreover, CuET interfered with the activation of the RPA-ATRIP-ATR-CHK1 repair pathway, by suppressing the ATR kinase despite of high replication stress, induced by ssDNA aggregates (Majera et al., 2020).

### Inhibition of Cancer Stem Cells (CSC)

Cancer stem cells (CSC) are highly resistant to several conventional chemotherapies and therefore, these cells are responsible for the cancer recurrence (Al-Hajj et al., 2003; Singh et al., 2004; Ginestier et al., 2007; Morgan et al., 2009; Fernando et al., 2015; Liu et al., 2021). ALDH belongs to an enzyme superfamily that catalyses aldehydes, the toxic metabolite of alcohol. It is crucial for the synthesis of molecules that play important role in cell proliferation, differentiation, and survival (Jackson et al., 2011; Pors and Moreb, 2014). Over a decade, the association between high ALDH activity and the cancer stem cell (CSC) phenotype has inspired scientists to develop specific ALDH inhibitors with greater clinical potential to effectively suppress CSCs and tumour progression (Jackson et al., 2011). The ability of DSF to induce apoptosis in breast CSCs is due to the inhibition of ALDH1 activity (Yip et al., 2011). DSF was found to enhance the cytotoxic effect of cisplatin by inhibiting the stemness of CSCs derived from breast cancer cell lines through the inhibition of expression of stemness-related transcription factors such as Sox, Nanog, and Oct and also by inhibiting the activity of ALDH in ALDH + stem-like cells (Liu et al., 2013; Yang et al., 2019). Guo et al. (2019) also demonstrated that DSF sensitized the ovarian cisplatin-resistant ALDH+ stem-like population to cisplatin treatment by suppressing the ALDH activity and inducing the apoptosis (Guo et al., 2019). DSF also reverses the resistance of testicular germ cell tumours towards cisplatin by inhibiting the ALDH activity (Schmidtova et al., 2019). It was found that supplementation of Cu with DSF augments its activity in reversing the chemoresistance of drug-resistant cancer cells (Liu et al., 2013). DSF-Cu complex supressed the CSCs in breast cancer by inhibiting the ALDH1 activity, supressing the CD44+/CD24− and CD49f+/CD24+ subpopulations and downregulating the phospho-STAT3, cyclin D1 and surviving of STAT3 signalling pathway (Kim et al., 2017). DSF-Cu complex also reversed the microtubule inhibitors resistance in A549/Taxol (Taxol-resistant cells) and KB/VCR (vincristine-resistant cells) cells by decreasing the activity of ALDH2 and inhibiting the expression of P-gp (glycoprotein P) which is highly expressed in microtubule inhibitors resistant cancer cells (Wang et al., 2018). DSF-Cu complex was found to be more efficient as compared to DSF alone in eliminating the multiple myeloma stem cells by inhibiting the ALDH activity and suppressing the stemness-related transcription factors such as Nanog, and Oct4 (Jin et al., 2018).

## Limitations of Disulfiram

Although DSF shows high *in vitro* toxicity in cancer cells, there was almost no positive clinical data published in cancer patients https://clinicaltrials.gov/ct2/results?term=disulfiram+AND+cancer&Search=Search). A summary of all the clinical trials using oral DSF is given in Table 2.

**TABLE 2:** Summary of clinical trials using disulfiram for cancer treatment.

Study Title	Tumour Type	Drugs	Dose	Status	Outcome	Identifier
						
Phase II Trial of Disulfiram With Copper in Metastatic Breast Cancer	Breast cancer (Metastatic)	Disulfiram/Copper Supplement	DSF: 400 mg p.o./d Cu: 2 mg p.o./d	Phase II, Recruiting	N/A	NCT03323346
Vinorelbine, Cisplatin, Disulfiram and Copper in CTC_EMT Positive Refractory Metastatic Breast Cancer	Breast Cancer (Refractory, HER2-VE)	Vinorelbine, Cisplatin, Disulfiram, Copper	DSF: 400 mg p.o./d Cu: 2 mg p.o./d	Phase II, Recruiting	N/A	NCT04265274
Disulfiram and Cisplatin in Refractory TGCTs	Germ cell tumour	Disulfiram, Cisplatin	DSF: 400 mg p.o./d	Phase II, Recruiting	N/A	NCT03950830
Disulfiram in Treating Patients With Glioblastoma Multiforme After Radiation Therapy With Temozolomide	Glioblastoma	Disulfiram/Copper, Gluconate, Temozolomide	DSF: 500 mg p.o./d; Cu: 6 mg p.o./d	Early Phase I, Completed	Max. tolerated dose = 500 mg/day	NCT01907165
Disulfiram and Copper Gluconate With Temozolomide in Unmethylated Glioblastoma Multiforme	Glioblastoma	Disulfiram/Copper Gluconate Temozolomide	DSF 125 mg p.o./twice daily; Cu: 2 mg p.o./twice daily	Phase II, Recruiting	N/A	NCT03363659
Disulfiram/Copper Combination In The Treatment of Newly Diagnosed Glioblastoma Multiform	Glioblastoma	Disulfiram/Copper Temozolomide	N/A	Phase II, Not Yet Recruiting	N/A	NCT01777919
Disulfiram in Recurrent Glioblastoma	Glioblastoma	Disulfiram/Copper Temozolomide	DSF 400 mg p.o./d; Cu: 2 mg p.o./d	Phase II/III, Completed	N/A	NCT02678975
Bioavailability of Disulfiram and Metformin in Glioblastomas	Glioblastoma	Disulfiram, Metformin	DSF: 200 mg p.o./twice/d Cu: 2.5 mg p.o./d	Early Phase I, Terminated	N/A	NCT03151772
Disulfiram/Copper With Concurrent Radiation Therapy and Temozolomide in Patients With Newly Diagnosed Glioblastoma	Glioblastoma	Disulfiram/Copper Gluconate	DSF: 125 mg, 250 mg, 375 mg or 500 mg p.p./d	Phase I/II, Recruiting	Low toxicity at 250 mg/day. Median follow-up of 12.3 mo, 1-yr PFS: 57%; 1-yr OS: 69%	NCT02715609
			Cu: 2 mg p.o./thrice daily			
Safety, Tolerability and Efficacy of Disulfiram and Copper Gluconate in Recurrent Glioblastoma	Glioblastoma (recurrent)	Disulfiram/Copper, Temozolamide	DSF/Cu: 80 mg p.o./thrice daily	Phase II, Completed	Low toxicity (1/23 with grade 3 elevated ALT); ORR 0/23; 14% with clinical benefit	NCT03034135
A Proof-of-concept Clinical Trial Assessing the Safety of the Coordinated Undermining of Survival Paths by nine Repurposed Drugs Combined With Metronomic Temozolomide (CUSP9v3 Treatment Protocol) for Recurrent Glioblastoma	Glioblastoma (recurrent)	Disulfiram Metronomic Temozolamide	250 mg p.o./d; 250 mg p.o. twice daily	Phase I, Active Not Recruiting	N/A	NCT02770378
Disulfiram and Chelated Zinc for the Rx of Disseminated Mets Mel That Has Failed First Line Therapy	Melanoma	Disulfiram and Zinc	N/A	Phase II, Completed	1 grade 3+ toxicity (confusional episode); ORR 0/12; 1 target lesion –27%	NCT02101008
Disulfiram Plus Arsenic Trioxide In Patients With Metastatic Melanoma and at Least One Prior Systemic Therapy	Melanoma (Metastatic)	Disulfiram	250 mg p.o. twice daily	Phase I, Terminated	N/A	NCT00571116
Disulfiram in Patients With Metastatic Melanoma	Melanoma (Stage IV)	Disulfiram	250 mg p.o. twice daily, escalated until maximum tolerated dose	Phase I/II, Completed	N/A	NCT00256230
Study of Disulfiram and Copper Gluconate in Patients With Treatment-Refractory Multiple Myeloma	Multiple Myeloma (Refractory)	Disulfiram, Copper Gluconate	N/A	Phase 1, Recruiting	N/A	NCT04521335
Initial Assessment of the Effect of the Addition of Disulfiram (Antabuse) to Standard Chemotherapy in Lung Cancer	Non-Small Cell lung Cancer	Disulfiram, Chemotherapy	N/A	PhaseII/III, completed	Benefit in PFS (5.9 vs 4.9 mo) and OS (10.0 vs 7.1 mo)	NCT00312819
Disulfiram-Copper Gluconate in Met Pancreas Cancer w Rising CA19-9 on Abraxane-Gemzar, FOLFIRINOX or Gemcitabine	Pancreatic Cancer (Metastatic)	Disulfiram/Copper Gluconate	N/A	Phase II, Recruiting	N/A	NCT03714555
Disulfiram and Chemotherapy in Treating Patients With Refractory Solid Tumors or Metastatic Pancreatic Cancer	Pancreatic Cancer (Metastatic, Recurrent)	Disulfiram, Gemcitabine	250 mg p.o./d, 28 days	Phase I, Recruiting	N/A	NCT02671890
A Phase Ib Study of Intravenous Copper Loading With Oral Disulfiram in Metastatic, Castration Resistant Prostate Cancer	Prostate Cancer (Metastatic Castrate-resistant)	Disulfiram/Copper Gluconate	DSF: 80 mg p.o./three times daily; Cu: 1, 3, 5, or 7 mg on cycle 1 days 1, 8, and 15	Phase I, Terminated	No grade > 3 toxicities; no effect on PSA; 64Cu-PET shows Cu-uptake in some metastases	NCT02963051
Study of Recurrent Prostate Cancer With Rising Prostate Specific Antigen (PSA)	Prostate Cancer (Recurrent)	Disulfiram	250 mg p.o./d, 28 days; 500 mg p.o./d, 28 days	Completed	Moderate tolerability (6/19 with grade 3); 5/19 (26%) patients with change in 5-methyl-cytosine; no effect on PSA levels	NCT01118741
Phase I Study of Disulfiram and Copper Gluconate for the Treatment of Refractory Solid Tumors Involving the Liver	Solid Cancer (Refractory, liver)	Disulfiram/Copper Gluconate	DSF: 250 mg p.o./d Cu: 2 mg p.o./d	Phase I, Completed	Well tolerated; no dose limiting toxicity; one stable disease	NCT00742911

This inefficiency of DSF could be attributed to the very short half-life of the currently available oral version of DSF in the blood stream which is approximately 2–4 min (Marselos et al., 1976; Eneanya et al., 1981; Johansson, 1992). Our previous findings indicated that the cytotoxic effect of DSF on cancer cells is executed in two phases: *1*) The instant ROS mediate damages induced by the reaction between DSF and Cu; *2*) A delayed killing induced by the end product, Cu-DDC (Butcher et al., 2018). After ingestion of the current oral version, DSF is rapidly reduced into two DDC molecules *via* the disulphide bond breakage in the gastrointestinal system and the bloodstream of the portal vein (Johansson, 1992). The DDC which is accumulated in the liver is still a highly reactive chelator of divalent metal ions, specifically Cu and Zn. But in the liver DDC is rapidly degraded into carbonyl disulphide and dimethylamine or undergoes methylation enzymatically to form S-methyl-DDC (Johansson, 1992; Butcher et al., 2018). As a result, DDC and all metabolites of DSF lose their active functional thiol groups and their ability to chelate with Cu or other metal ions is compromised. Surprisingly this process does not inhibit the anti-alcohol dependency activity of DSF as the metabolization of DSF takes place in the liver, which is the site of ethanol breakdown and hence it is able to successfully inhibit ALDH activity (Marselos et al., 1976; Eneanya et al., 1981; Petersen, 1992; Lewis et al., 2014). Whereas in the case of cancer, the reaction of DSF and Cu must take place inside or adjacent to cancer cell but mostly the target tissue where tumour is located are at a distant site. Hence the active sulfhydryl groups in DDC are indispensable for the chelation reaction between DDC and Cu and formation of Cu-DDC complex (Liu et al., 2014; Butcher et al., 2018).

**FIGURE 4:**
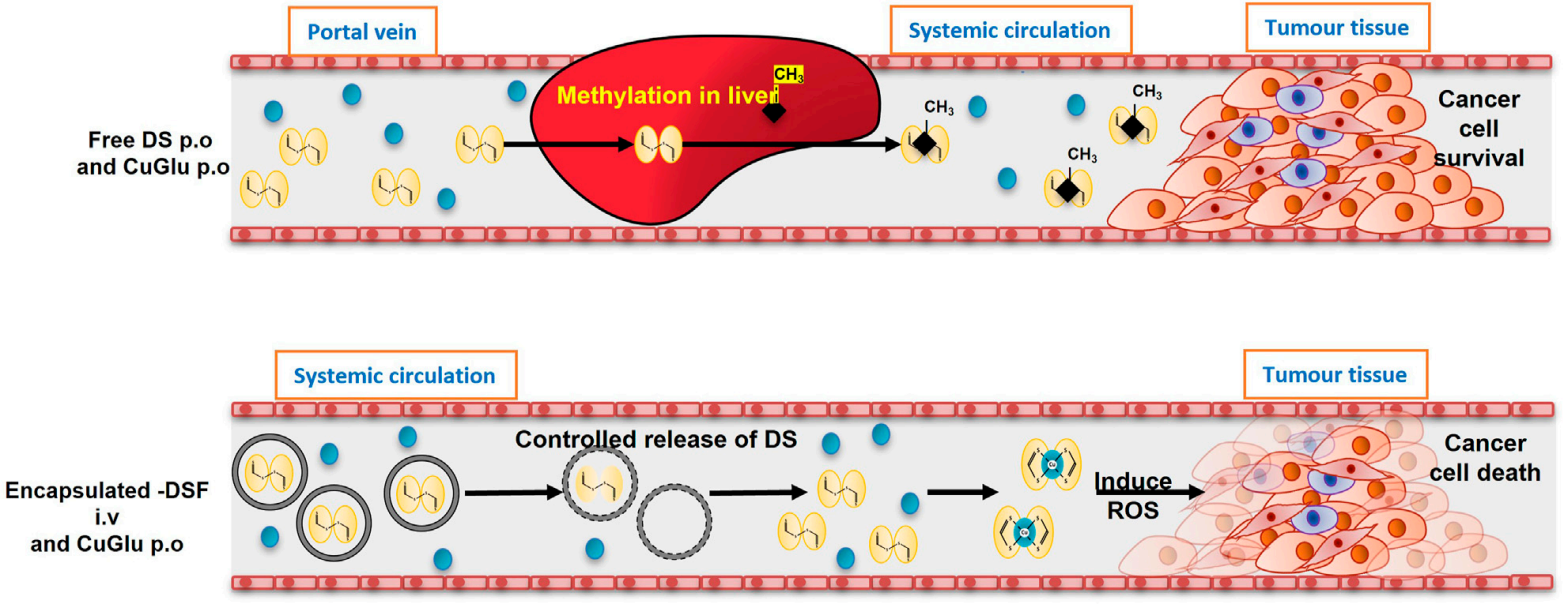
The models of oral and intravenous administration of DS. A. After oral administration, DS will be delivered *via* portal vein and enriched in liver in which it is instantly methylated. The methylated DS inhibits ALDH and remains its antialcoholism function. Because the thiol group is blocked, its anticancer activity is completely abolished. B. In the new intravenous formulation of DS, the thiol groups in DS are protected. After delivered to cancer tissues, DS chelates copper to generate ROS inducing apoptosis. The end product, DDC-Cu, can penetrate into cancer cells and trigger apoptosis, as well (Tawari et al. Toxicol Res 2015; 4:1439). This hypothesis explained the discrepancy of anticancer activity of DS in the lab and in clinic and the need to develop intravenously applicable nano-encapsulated DS, e.g. liposome and PLGA encapsulated DS.

Therefore, we hypothesised that using nano-drug-delivery system to protect the sulfhydryl groups in DDC will overcome the discrepancy between the anticancer activity of DSF in laboratory and clinic and pave the path between “bench and bed.” Based on this hypothesis, our group first encapsulated DSF into liposomes and demonstrated significantly improved anticancer efficacy in mouse models (Liu et al., 2014). Development of novel formulations of DSF using nano-drug delivery strategies will be of significant clinical importance in translation of DSF into cancer treatment (Figure 4).

## Nanotechnology-based Formulation Strategies for Delivery of Disulfiram

The use of nanotechnology, a booming field in the last decade, could provide us with a cutting-edge drug delivery system for translating DSF into cancer treatment. There are various methods such as adjusting the formulation, structural modification, or encapsulation techniques, all of which aims at increasing the bioavailability of the interested compound. Nanoencapsulation involves the use of biodegradable compounds such as liposomes, polymers, polymeric micelles or protein (albumin) particles to encapsulate the drug of interest (Shi et al., 2017). Several FDA-approved formulations of nano encapsulated conventional anticancer drugs such as albumin nanoparticle Nab-paclitaxel (Abraxane™) and liposome encapsulated doxorubicin are currently used in the clinics. A summary of various strategies developed by different groups for delivering DSF is given in Table 3.

**TABLE 3:** Formulation strategies used for delivery of disulfiram for cancer treatment.

Formulation	Components and technique used	Cancer indication	Cell lines/models	Study outcome	Reference
*Polymers*					
DSF encapsulated mixed nanoparticles	mPEG5000-PCL5000, PCL5000, MCT and DSF – High pressure homogenization method	Mouse hepatocellular adenocarcinoma	H22 xenograft tumor model	• Increased Drug loading and stability	Zhuo et al. (2018)
				• Higher degree of tumor necrosis and tumor inhibition in animal models	
DSF encapsulated PLGA nanoparticles	PVA, PLGA, DSF Nanoprecipitation method	Breast Cancer	MCF7 cell lines	• Significantly enhanced stability when compared to free drug in serum solution (10% FBS)	Fasehee et al. (2017)
				• Enhanced cytotoxicity compared to free drug	
Folate receptor targeted PLGA-PEG nanoparticles encapsulated with DSF	PLGA, (PEG)-bis amine, PVA, Folate, DSF Nanoprecipitation method	Breast cancer	MCF7 and 4T1 cell lines, BALB/c mice models	• Showed a higher degree of cytotoxicity compared to free drug and non-folate formulations	Fasehee et al. (2016)
				• Significant decrease of tumor size in mouse models	
Combination of polyethylene glycol-cisplatin complex and DSF encapsulated nanoparcticles	mPEG-PLGA/PCL, PGA, DSF, Cisplatin	Lung cancer	A549 and A549DDP cell lines. BALB/c mouse model	• synergistically enhanced the cytotoxic effect of cisplatin *in vitro*	Song et al. (2016)
				• The combination effectively inhibited the tumor growth and displayed excellent safety in mouse models	
PLGA encapsulated DSF Nanoparticles	PVA, PLGA, DSF. emulsion-solvent evaporation method	Lung cancer	A549 cell lines	• enhanced half-life in serum	Najlah et al. (2017)
				• Higher cytotoxicity compared to free DSF *in vitro*	
Brain tumor-penetrating disulfiram nanoparticles	mPEG, PLGA, DSF. emulsion-solvent evaporation method	Brain cancer	T98G and DAOY cell lines and Female CD-1 mice, female athymic nude (nu/nu) mice, and the triple immune-deficient NCG mice models	• Enhanced delivery of DSF to the brain tumor site and increased stability	Madala et al. (2018)
				• Potent cytotoxic and anti-clonogenic activities	
*Lipid based nanoparticles*					
Liposome encapsulated formulation of DSF	PC, CHOL, PG Thin film hydration to form multilamellar vesicles subject to size reduction -100–300 nm	Breast Cancer	MCF7, MDA-MB-231, T47D S180 mouse sarcoma models - BALB/c mice. MDA breast cancer models -CD1 Nu/Nu mice	• Selective targeting of cancer cells, induction of apoptosis	(EP2648709B1, 2011; Liu et al., 2014)
				• Enhanced efficacy of conventional anticancer drugs	
				• Target cancer stem cells- reduction in CSC markers and clonogenicity	
				• Protection of thiol group *in vivo*, react with Cu to exert anticancer activity in mouse models of sarcoma and breast cancer	
Co-encapsulated liposomal formulation of Doxorubicin and DSF	DSPC, CHOL, mPEG2000-DSPE. Thin film hydration method	Breast cancer	MCF7, MDA-MB-231	• Decrease in Pgp expression and reversal of Pgp mediated drug resistance	Rolle et al. (2020)
				• Enhanced efficacy of DOX	
DSF entrapped vitamin E-TPGS-modified PEGylated nanostructured lipid carriers	Lecithin, Precirol^®^ ATO, Labrafac Lipophile WL134, DSF. Emulsification ultrasonication method	Breast cancer	MCF7, 4T1, BALB/C mouse models	• Enhanced long term stability	Banerjee et al. (2019)
				• Higher toxicity compared to free drug *in vitro*	
				• Significant increase in tumor inhibition with no toxicity *in vivo*	
PEGylated Liposome Encapsulating DSF	HSPC, DPPC, CHOL, DSPE-PEG2000, DSF. Ethanol-based proliposome technology	Colorectal cancer	H630-WT and H630-R10	• Significantly improved serum stability	Najlah et al. (2019)
				• Cytotoxic to colorectal cancer cell lines in presence of copper *in vitro*	
Micelle based nanoparticles					
pH triggered polymeric micelles for the co-delivery of paclitaxel/disulfiram	Methoxy PEG-b-PLL, DMA, PTX and DSF. Nanoprecipitation method	Breast Cancer	MCF7 and MAF7/ADR	• Enhanced internalization of nanoparticles into tumor cells	Huo et al. (2017)
				• Inhibition of Pgp transport function	
DSF encapsulated micelles	mPEG5k-b-PLGA2k/PCL3.4 k/MCT, DSF solvent diffusion method	Liver cancer	H22 xenograft mouse model	• Increased stability and bioavailability in plasma	Miao et al. (2018)
				• Enhanced tumor inhibition in mouse model	
DSF loaded redox-sensitive shell crosslinked micelles	SMA micelles were crosslinked by adding cystamine dihydrochloride and sodium bicarbonate	Breast cancer	4T1	• Significant tumour inhibition	Duan et al. (2014)
				• Increased Stability	
Other Nanoparticle delivery systems					
DSF- loaded magnetic mesoporous silica nanoparticles	PEI-FA, Fe3O4@mSiO2 Np’s, DSF Thermal decomposition and Sol gel reaction method	Breast Cancer	MCF7	• Exhibited selective toxicity to tumor cells	Solak et al. (2021)
				• Highly cytotoxic in the presence of copper	
Cu(DDC)2 and regorafenib encapsulated BSA *nanoparticles*	Denaturing of BSA by urea to induce *nanoparticle* formulation	Glioma	H22	• Improved stability and increased efficacy against resistant xenografted tumors	Zhao et al. (2018)
DSF loaded gold nanorods	Seed solution preparation and surface PEG modification	Breast Cancer	MCF-7	• Improved circulation time, increased tumor accumulation, tumor shrinkage *in vivo*	Xu et al. (2020a)
Cyclodextrin Cu(DDC)2	SBE-CD and HP-CD were the cyclodextrins used	Breast cancer	MDA-MB231	• Stability for 28 days, retained anti-cancer properties of Cu(DDC)2	Said Suliman et al. (2021)

Polymeric nanoparticles are simple in design and encompass extensive structural diversity, outstanding bio-compatibility and stability (El-Say and El-Sawy, 2017). One of the best examples of polymeric nano encapsulation is PLGA. It was demonstrated that PLGA encapsulation shielded DS from breakdown in the bloodstream and transported the intact DSF to cancer tissues (Wang et al., 2017b). The efficacy of PLGA-based DSF nanoparticles was demonstrated in a wide range of cancers using xenograft mouse models of hepatocellular carcinoma, lung cancer, ovarian cancer, and breast cancer (McConville et al., 2015; Fasehee et al., 2016; Wang et al., 2017a; Fasehee et al., 2017; Najlah et al., 2017). In addition, polymeric nanoparticles can be modified extensively to add functional groups that can be targeted for delivery to a tissue of interest. For example, a folate receptor targeted PEG-PLGA was shown to deliver and enrich DSF specifically to breast cancer tissues while avoiding degradation of DSF in the bloodstream (Fasehee et al., 2016). Our group developed DSF loaded PLGA nanoparticles through emulsion-solvent evaporation method with excellent entrapment efficiency and better drug loading. The nanoparticles were in the size range of ∼136 nm and had significantly extended the half-life from 2 min to 7 h in serum. The encapsulated DSF also demonstrated a significant synergistic cytotoxicity in combination with 5-FU or sorafenib and inhibited the growth of cancer stem cell populations in liver cancer models and reduced the metastatic activity and recurrence in mouse HCC xenograft and metastatic models (Wang et al., 2017b).

Liposomes which are artificial spherical vesicles prepared from naturally derived phospholipids are the most widely investigated and well-established drug delivery carriers used in clinics to treat various cancers due to their biocompatibility, specifically being non-immunogenic, non-toxic, and biodegradable. In addition, liposomes serve as sustained release systems providing protection against degradation and increased the time in circulation (Moosavian et al., 2021; van der Koog et al., 2021). In this sense, our group recently demonstrated that liposome encapsulated DSF a relatively extended half-life in serum and a significantly increased anticancer efficacy in *in vitro* and *in vivo* breast cancer mouse models (Liu et al., 2014).

Considering the hydrophobic nature of DSF, liposome-based delivery system would be an exciting prospect. Our group has developed liposome encapsulated DSF which shows significantly improved anticancer efficacy in breast cancer and sarcoma mouse models (EP2648709B1, 2011; Liu et al., 2014). Recently, we also encapsulated DSF into a PEGylated liposomal formulation of DSF using ethanol-based proliposome technique followed by high-pressure homogenization with significantly improvement in the stability of DS in serum and effective cytotoxicity to colorectal cancer cell lines in the presence of Cu *in vitro* (Najlah et al., 2019). Another group has developed an encapsulated formulation of DSF and doxorubicin which reduced the expression of P-gp and increased the efficacy of doxorubicin in breast cancer cells (Rolle et al., 2020). Banerjee et al. (2019) has shown that a formulation of nanostructured lipid carriers (NLC) encapsulated DSF modified with d-α-tocopheryl polyethylene glycol 1000 succinate (vitamin E-TPGS) showed an enhanced uptake in breast cancer cell lines and significantly reduced the tumour size and enhanced tumour suppression in xenografted mouse models (Banerjee et al., 2019).

Micelles have unique properties such as low molecular weight, better entrapment and enhanced delivery in tumour sites and are very suitable for a hydrophobic drug like DSF. They can easily penetrate the tumour tissue in comparison to other nanoparticles (Deshmukh et al., 2017). Our group developed a Pluronic micelle formulation to deliver DSF for triple negative breast cancer treatment. However, the main drawback of using micelles for DSF is their stability. Duan et al. developed a micelle cross-linked with redox sensitive shells that delivers DSF in reduced environments of the tumours (Duan et al., 2014). Another modified version of micelles using PEG and poly-lysine copolymers blocks were also used for delivery of DSF and paclitaxel together for tackling multidrug resistance and P-gp inhibition in breast cancer models and resulted in enhanced uptake of paclitaxel by breast cancer cells (Huo et al., 2017).

There are also more unique methods of delivering DSF; for example, magnetic mesoporous silica nanoparticles were used to specifically deliver DSF to breast cancer cells (MCAF) *in vitro* (Solak et al., 2021). DSF has also been loaded onto gold nanorods with a reported a drug loading content of up to 23.2% (Xu et al., 2020a). The Au-DSF product was able to effectively increase ROS and induce apoptosis in breast cancer cells *in vitro*.

## Delivery of DDC Complexes with Macromolecules

Albumin is a promising macromolecular drug carrier as it is biocompatible, biodegradable, non-immunogenic and soluble in water to allow for injection. In addition to this, it is possible to modify the surface of albumin nanoparticles for targeting. Previously, nanoparticle albumin bound technology has been used to solubilize the hydrophobic cancer drug paclitaxel (Abraxane) which is now a first line drug for PDAC (Stinchcombe, 2007). Since the complexes Cu(DDC)_2_ and Zn(DDC)_2_ are hydrophobic; nanoparticle albumin bound technology may be the key to develop an injectable, long circulating formulation.

Cu(DDC)_2_ and Regorafenib (Rego) were successfully encapsulated in bovine serum albumin (BSA) nanoparticles *via* denaturation of BSA by urea (Zhao et al., 2017). The formulation targeted secreted protein acidic and rich in cysteine (SPARC) receptors. Furthermore, mannose receptors were targeted by modifying the surface of the BSA nanoparticles. It had enhanced anti-cancer efficacy in an animal model bearing resistant tumours *via* apoptosis, upregulation of intracellular ROS, anti-angiogenesis and tumor-associated macrophage repolarization. These nanoparticles could also effectively cross the blood brain barrier to deliver Cu(DDC)_2_ and Rego to treat gliomas in mice (Zhao et al., 2018). The option to selectively target cancer cells by modifying the surface of the BSA nanoparticles could be vital when delivering DDC already complexed with Cu compared with DSF alone. DSF targets cancer cells due to their increased levels of Cu. On the other hand, a Cu(DDC)_2_ complex may have increased toxicities on normal cells. Another suitable macromolecule carrier for DSF is Cyclodextrans (CDs) which are cyclic oligosaccharides that can form truncated-cone-shaped macrocyclic molecules that possess hydrophobic cavities. These cavities can solubilize hydrophobic drugs and Suliman et al. (2021) developed a CD formulation of Cu(DDC)_2_ and demonstrated stability for 28 days with enhanced cytotoxicity against resistant breast cancer cell lines (Said Suliman et al., 2021).

Together, all the above evidence has indicated that using nanotechnology-based formulation strategies can significantly improve the half-life, stability, bioavailability and it is possible to achieve delivery of DSF at therapeutic doses in cancer patients.

## Conclusion

The success of platinum drugs has led to the search for metal complex-based anticancer therapeutics. The search for novel metallodrugs or metal complexes that target cancers is still a field of high interest in the pharmaceutical sector. The fundamental role of Cu in aiding cancer progression and the dysregulation of Cu homeostasis in cancers has been well documented in both preclinical and clinical settings. With Cu as an emerging novel target, there has been major advancements in the development of Cu coordination complexes as anti-cancer therapeutics. Use of Cu chelators and Cu ionophores have yielded encouraging anticancer activities in many preclinical models. Particularly many drugs that are used for other indications have been identified and repurposed as Cu coordination drugs, not only to achieve better antitumour efficacy, but also to enhance the efficacy of first line anticancer drugs such as platins.

Among many repurposed metal chelator drugs, DSF has a great promise and potential to be used as an anticancer agent. With its anti-angiogenic potential, and the ability to target CSCs and synergistically potentiate many first line conventional drugs, DSF could provide new opportunities to develop effective combination therapeutic strategies. DSF also has a great potential be an effective immunomodulatory drug by regulating NF-kB signalling or by reducing PDL-1 expression by Cu chelation. In this review we have summarised the effectiveness of DSF as an anticancer agent, which applies to a wide range of cancers. Despite several clinical trials, no reliable efficacy of DSF as an anticancer drug for tumours has been observed due to the lack of biological availability and short half-life of the currently available oral DSF. Therefore, the development of an appropriate formulation is critical for the successful translation of DSF into cancer indication. Here we summarized different delivery strategies that have been developed for DSF-based cancer therapy. Most of the identified or developed drug delivery systems for DSF are at the proof-of-concept stage either in *in vitro* studies or *in vivo* pre-clinical animal models. Although many of these delivery strategies demonstrated great potentials for DSF as an anticancer agent, these delivery systems must be extensively evaluated with further studies to gather complete preclinical information such as safety, toxicology, pharmacokinetic and optimal physicochemical properties which are crucial to facilitate clinical translation.

Although many different delivery strategies using novel materials indicated additional benefits, the use of FDA approved excipients could minimize regulatory hurdles and considerably reduce the preclinical development time. Moreover, the development of encapsulation strategies should be suitable for scale-up production for manufacturing and commercialization. In addition to these technical issues, there are many regulatory and marketing hurdles for commercialization of DSF based anticancer product, predominantly due to the lack of patent protection on DSF.

Therefore, pharma companies do not show much interest to invest in clinical trials for DSF-based anticancer therapeutics. The development of novel formulations of DSF together with appropriate strategies for exploitation of regulatory guidelines, would lead not only to the development of intellectual properties and enhanced anticancer efficacy, but also will lead to the successful development and clinical translation of DSF as an anticancer product for commercialization.
